# Artificial Intelligence and Digital Pathology: Technological Transformation and Strategic Impact in Clinical Research and Medical Affairs

**DOI:** 10.3390/life16081346

**Published:** 2026-08-16

**Authors:** Carmela Baviello, Daniela Maria Capuano, Roberto Verna

**Affiliations:** 1 Research and Training Center for Health and Well-Being, University Guglielmo Marconi, via Plinio 44, 00193 Roma, Italy; carmelabaviello@gmail.com (C.B.); capuano.accademia@gmail.com (D.M.C.); 2 Academy for Health and Clinical Research, University Guglielmo Marconi, via Plinio 44, 00193 Roma, Italy; 3 World Association of Societies of Pathology and Laboratory Medicine, University Guglielmo Marconi, via Plinio 44, 00193 Roma, Italy

**Keywords:** Artificial Intelligence, digital pathology, clinical research, medical affairs

## Abstract

The progressive integration of Whole Slide Imaging (WSI) technology and Artificial Intelligence (AI) architectures is driving a structural transformation in pathology and precision oncology. This structured critical review analyzes and systematizes the impact of this technological transition along two fundamental operational dimensions of the modern biopharmaceutical industry: pre-registration Clinical Research and post-launch strategies governed by Medical Affairs. The first section explores how computational pathology is improving efficiency and reducing risk in drug development. Replacing analog visual assessment—intrinsically subject to inter-observer and intra-observer variability—with quantitative algorithms for cellular classification and segmentation enables optimization of patient recruitment in clinical trials, reducing screening failure rates. This review also examines the emerging role of Spatial Biology in extracting complex topological metrics from the Tumor Microenvironment (TME) and the use of AI for the objective and auditable quantification of critical surrogate endpoints, such as Pathological Complete Response (pCR), while acknowledging that algorithmic precision remains sensitive to pre-analytical variables and dataset biases. In the second section, the study investigates the strategic evolution of Medical Affairs, acting as a vital scientific communication and translational bridge between the complexity of Data Science and clinical hospital practice. Challenges related to AI adoption by clinicians are examined, emphasizing the importance of educational programs based on Explainable AI (XAI) to overcome the cognitive limitations of the black-box paradigm and the complex regulatory validation pathway for Software as a Medical Device (SaMD) under the stringent European IVDR framework—supported by an analysis of historical regulatory benchmarks such as the Paige Prostate case. The paper also explores the potential of AI in the large-scale generation of Real-World Evidence (RWE), applied to the creation of synthetic control arms in pharmacoeconomic settings. In conclusion, the study highlights that the diagnostic algorithm has ceased to be merely a laboratory support tool and has become a strategic asset and an integral adjunct to therapeutic decision-making. Overcoming current challenges related to data privacy through Federated Learning architectures, together with the imminent transition toward Foundation Models, foreshadows a fully data-driven healthcare ecosystem, making continuous skills development (digital upskilling) an essential requirement for professionals in the biopharmaceutical sector.

## 1. Introduction

### 1.1. The Legacy of Analog Pathology and the Imperative for Change

For more than a century, pathology has been based on a methodological paradigm that has remained largely unchanged. The visual observation of formalin-fixed paraffin-embedded (FFPE) tissue samples through optical microscopy remains the gold standard for oncological diagnosis. However, despite having guided the progress of modern medicine, this approach presents structural limitations. The diagnostic process is closely linked to the visual perception of the individual operator, making the assessment qualitative in nature and intrinsically subjective.

Several multicenter concordance studies have extensively documented the problem of “inter-observer” and “intra-observer” variability. Diagnostic discrepancies may occur between different specialists or even with the same pathologist at different times when analyzing the same histological specimen [1]. This interpretative heterogeneity carries critical implications in the current clinical landscape. The assessment of morphologically complex grading systems, such as the Gleason score in prostate cancer [2], or the quantification of immunohistochemical biomarkers, now requires an absolute degree of precision. In the era of precision medicine, the difference between 1% and 0% expression of receptors such as PD-L1 directly determines a patient’s eligibility for targeted and high-cost immunotherapies, although specific thresholds and scoring systems heavily depend on the tumor type and the validated assay used [3]. Faced with therapeutic decisions of such significance, traditional visual estimation reveals its inherent limitations, making the transition to quantitative and reproducible assessment systems a clinical necessity that can no longer be postponed.

### 1.2. The Advent of Digitalization: The Transduction of Tissue

Over the last decade, the progressive clinical validation of Digital Pathology has brought about a decisive turning point in both diagnostics and research. The introduction of Whole Slide Imaging (WSI) enables the automated scanning of physical slides, transforming them into ultra-high-resolution (gigapixel) digital files and significantly changing laboratory workflows [4]. This is not merely a matter of dematerializing physical archives or simply acquiring an image. This transition transforms the underlying information itself: the complex cellular architecture of biological tissue is converted into a matrix of computable and quantifiable data. It is within this convergence of tissue biology and computer science that Artificial Intelligence (AI) finds its place. By leveraging Convolutional Neural Networks (CNNs) and Deep Learning architectures applied to computer vision, it is now possible to analyze billions of pixels in extremely short timeframes. These systems go beyond merely supporting human observation: they can identify non-obvious morphological patterns, objectively quantify protein expression, and extract prognostic and predictive “sub-visual” signatures that are inaccessible to the optical resolution of human pathological assessment [5,6].

### 1.3. The Pharmaceutical Ecosystem and the Objective of This Review

Although digital pathology was originally developed to optimize primary diagnostics in hospitals, its most significant impact is now being observed within the biopharmaceutical industry. While driven by advances in precision medicine, this sector simultaneously faces the so-called “Eroom’s Law,” a trend highlighting the progressive increase in Research and Development (R&D) costs alongside a general decline in the approval of new molecular entities [7]. Today, the development of targeted therapies, antibody-drug conjugates (ADCs), and immunotherapies requires increasingly rigorous and phenotypically accurate patient selection criteria. Consequently, the integration of Artificial Intelligence into histopathological analysis is no longer a theoretical prospect but a necessary resource for ensuring the methodological standardization required by global multicenter clinical trials. At the same time, it provides innovative strategic and educational tools for pharmaceutical medical departments. In light of this technological and scientific context, our objective was to analyze and systematize the impact of Digital Pathology and Artificial Intelligence along two central operational dimensions of the drug life cycle: Clinical Research (with a focus on pre-registration development phases) and Medical Affairs (in the areas of scientific communication, regulatory compliance, and post-launch evidence generation).

## 2. Methods: Search Strategy and Selection Criteria

To ensure the transparency, rigor, and reproducibility of this critical review, a structured literature search was conducted following established methodological guidelines. The objective was to comprehensively map the intersection of Artificial Intelligence, Digital Pathology, Clinical Research, and Medical Affairs within the biopharmaceutical ecosystem.

### 2.1. Data Sources and Search Period

A comprehensive literature search was performed across two primary electronic databases: PubMed/MEDLINE and Scopus. To capture the rapid technological evolution from early digital pathology milestones to the latest Generative AI architectures, the primary literature search focused on articles published between 2018 and 2024, while foundational historical benchmarks and regulatory guidelines were retrieved without temporal restriction. Furthermore, grey literature—specifically official regulatory documents and guidelines from the U.S. Food and Drug Administration (FDA) and the European Medicines Agency (EMA)—was systematically manually searched and retrieved to assess the regulatory frameworks surrounding Software as a Medical Device (SaMD).

### 2.2. Boolean Search Strings

The search strategy employed a combination of Medical Subject Headings (MeSH) and free-text keywords, aggregated using Boolean operators (AND, OR). The core search string was structured across three main conceptual domains:

1. (“Digital Pathology” OR “Whole Slide Imaging” OR “WSI” OR “Computational Pathology”) AND
2. (“Artificial Intelligence” OR “Deep Learning” OR “Machine Learning” OR “Convolutional Neural Networks” OR “Foundation Models” OR “Large Language Models”) AND
3. (“Clinical Trials” OR “Medical Affairs” OR “Drug Discovery” OR “Precision Oncology” OR “Biomarker” OR “Companion Diagnostics” OR “Synthetic Control Arms”).

### 2.3. Inclusion and Exclusion Criteria

To maintain a high standard of evidence, the inclusion criteria were strictly defined. Selected articles had to be: (1) peer-reviewed original research, critical reviews, or meta-analyses; (2) directly related to the application of AI in histopathology for oncology; (3) focused on translational implications, clinical trials, or regulatory and medical affairs perspectives; and (4) published entirely in the English language. Conversely, the exclusion criteria encompassed: (1) studies focusing solely on non-oncological pathologies; (2) articles lacking clinical or pharmaceutical translational relevance (e.g., purely theoretical computer science papers without clinical validation); (3) non-peer-reviewed preprints, conference abstracts without full text, and editorials lacking empirical data.

### 2.4. Study Selection Process

The initial database search yielded an extensive pool of records. After the automated removal of duplicates, the initial screening was conducted by evaluating titles and abstracts to exclude irrelevant publications. Subsequently, a full-text critical assessment of the remaining articles was performed to extract the most robust data. To avoid simply listing individual studies, the final selection prioritized landmark publications that provided multicenter validation, addressed persistent challenges (e.g., domain shift, dataset bias), or introduced disruptive architectural shifts (e.g., the transition from supervised CNNs to self-supervised Vision Transformers), ensuring a synthesized and critical overview of the current literature landscape.

## 3. Fundamentals of Digital Pathology and Artificial Intelligence

### 3.1. Whole Slide Imaging (WSI): The Architecture of Histopathological Big Data

The methodological prerequisite for applying Artificial Intelligence to pathology is the conversion of biological specimens into a processable digital format. In the past, telepathology was limited to acquiring individual microphotographs using cameras mounted on microscopes; this approach captured only specific areas of interest (the so-called Fields of View), inevitably sacrificing the tissue’s overall morphological context. Today, the transition toward digital diagnostics is based on Whole Slide Imaging (WSI) technology. Modern WSI scanners are advanced mechatronic systems that employ optical sensors (CCD or CMOS) and calibrated illumination systems to automatically acquire the entire surface of a glass slide. Acquisition is performed at typical magnifications of 20× or 40×, ensuring spatial resolutions of approximately 0.5 or 0.25 micrometers per pixel, respectively. The process relies on microscopic alignment and the subsequent software fusion (stitching) of thousands of individual frames or linear strips [8]. Furthermore, to manage cytological specimens in which cells tend to overlap in three-dimensional clusters, next-generation systems utilize Z-stacking, capturing multiple focal planes to simulate the depth focusing normally achieved using the microscope’s fine focus adjustment [9]. From a computer engineering perspective, WSI fully places histopathology within the realm of Big Data, surpassing even advanced radiology in terms of information density. A single slide scanned at 40× may contain several billion pixels, generating raw image files ranging from 1 to 5 gigabytes each. For a hospital or a Contract Research Organization (CRO) managing tens of thousands of slides per year, storage requirements rapidly scale into the petabyte range, posing significant challenges in terms of server infrastructure and bandwidth [10]. To enable zooming and panning through files of this size without the latency that would hinder diagnostic workflows, WSI software does not load the entire image into RAM. Instead, it uses a data architecture based on a “pyramidal format” [11]. The original image at maximum resolution forms the base of the pyramid; the system then generates progressively lower-resolution versions, up to a thumbnail positioned at the top. During on-screen navigation, the rendering software retrieves from the server only the individual tiles corresponding to the spatial coordinates and zoom level requested at that moment by the operator or the algorithm.

### 3.2. Algorithmic Evolution: From Machine Learning to Deep Learning

Within Digital Pathology, the reference computer science discipline is Computer Vision. Over the past decade, methods for extracting clinically relevant information from histological images have undergone a clear evolution. Initially, computational pathology systems relied on traditional Machine Learning (ML), using models such as Support Vector Machines or Random Forests. These approaches depended on a process known as feature engineering. Researchers had to translate the morphological parameters that the algorithm was expected to measure into mathematical formulas—for example, nuclear roundness or the nucleus-to-cytoplasm ratio [12]. This top-down approach soon revealed its limitations, proving too rigid to capture the extreme heterogeneity and complex structural abnormalities that characterize tumor tissues in clinical practice. Overcoming this limitation coincided with the adoption of Deep Learning (DL), particularly Convolutional Neural Networks (CNNs). The primary innovation of CNNs lies in representation learning: the human operator no longer defines which features to search for. By feeding the network with millions of image fragments (patches) derived from WSI, the algorithm autonomously learns to extract and hierarchically organize features through its hidden layers. During training, superficial layers specialize in detecting basic elements (such as lines and gradients), while deeper layers aggregate this information to recognize complex architectures, such as aberrant glandular formations or nests of apoptotic cells [6]. The current frontier of methodological research is the transition from Supervised Learning—which requires labor-intensive manual annotation of every region of the slide by a pathologist—to Multiple Instance Learning (MIL). MIL is a form of weakly supervised learning in which the algorithm is provided with the entire slide image associated only with a global label (for example, mutational status or patient survival). Through mathematical attention mechanisms, the neural network learns autonomously to identify which tiny regions within the giant slide are responsible for the clinical outcome, dramatically reducing data preparation times [13].

### 3.3. Taxonomy of Applications: Visual Tasks in Research and Diagnostics

Within clinical and experimental workflows, Deep Learning architectures (such as ResNet or U-Net) are designed to solve specific visual problems (tasks). The main applications, as systematized in the literature [14], can be divided into four broad categories:

Whole-Slide Classification: The algorithm analyzes the image as a whole to assign a global diagnosis (e.g., “Benign” vs. “Malignant”). More advanced models can further subclassify tumors, distinguishing, for example, adenocarcinoma from squamous cell carcinoma solely by observing the morphological patterns of routine staining, without the need for ancillary tests.

Object Detection (Detection and Localization): In this case, the model identifies and delineates specific discrete entities using precise spatial coordinates (bounding boxes). This is the preferred approach for the automated counting of mitotic figures or the identification of isolated lymph node micrometastases, reducing human error associated with natural visual fatigue.

Semantic and Instance Segmentation: This is the most computationally complex operation, requiring the assignment of a class to every individual pixel in the image. Semantic segmentation divides the slide into macro tissue regions (separating stroma from epithelium or necrotic areas), while instance segmentation delineates the exact boundaries of adjacent or overlapping individual cells, enabling advanced spatial analyses.

IHC Quantification and Scoring: This task applies to immunohistochemical (IHC) specimens to evaluate the expression of target proteins (such as ER, HER2, and Ki-67). The algorithm objectively detects chromogen intensity, providing an exact count of positive cells relative to the total and overcoming the natural visual approximations of human interpretation, which tends to estimate expression in percentage intervals.

### 3.4. The Major Barrier: Pre-Analytical Variables, Domain Shift, and Interoperability

Despite the high performance demonstrated in vitro, the translation of AI algorithms into real-world clinical practice faces obstacles related to the pre-analytical phase. The visual appearance of tissue under the microscope is sensitive to countless physicochemical variables: cold ischemia time, type of fixative, microtome section thickness, and fluctuations in staining protocols (Hematoxylin-Eosin), including variations arising from different reagent batches [15]. These variables introduce the problem of Domain Shift. An algorithm trained exclusively on perfectly standardized slides from a leading research center (the source domain) will almost certainly experience a decline in accuracy when tested on images from a peripheral laboratory (the target domain), whose slides may exhibit different color balances or contrast levels [16]. To enhance model robustness, developers employ advanced techniques. Among these, Color Normalization stands out, often implemented through Generative Adversarial Networks (GANs), which map and standardize the color spectrum of incoming images to a reference profile. Another essential technique is Data Augmentation, which artificially alters training data (by modifying brightness, contrast, or saturation) to force the network to ignore superficial color variations and base its predictions solely on cellular topology and morphology [17]. Regulatory agencies often require site-specific calibration or retraining of the AI algorithm on-site, rather than relying solely on computational color normalization.

Finally, large-scale adoption depends on the interoperability of IT systems. The gradual transition away from proprietary formats (vendor lock-in) tied to individual scanner manufacturers, in favor of the universal DICOM-WSI (Digital Imaging and Communications in Medicine for Whole Slide Imaging) standard, is laying the infrastructural foundation for seamless and vendor-agnostic integration of AI software into hospital workflows [18]. A comprehensive summary and critical analysis of the pivotal AI architectures, their clinical applications, and remaining structural challenges discussed in this field are provided in Table 1.

## 4. The Impact of Digital Pathology on Clinical Research (Clinical Trials)

The development, clinical validation, and regulatory approval of a new oncology drug represent one of the most complex challenges in biomedicine, frequently requiring investments exceeding one billion dollars and development timelines spanning a decade. In this context, characterized by a high rate of clinical attrition, the digitization of histopathology offers a notable risk-mitigation strategy. By transforming tissue biology from an analog finding into a true quantitative asset, computational pathology enables the optimization of critical phases of clinical studies, particularly in the complex Phase II and Phase III trial settings [22].

### 4.1. Optimization of Recruitment and Reduction in “Screening Failures”

The failure of many late-phase clinical trials, especially in the field of targeted therapies, is often attributable to the biological heterogeneity of the recruited cohort. Modern oncology therapies require patient inclusion to be based on the objective and measurable expression of specific tissue biomarkers. However, traditional optical microscopy interpretation is a semi-quantitative cognitive process inherently exposed to biases related to visual fatigue, differences in training, and the unavoidable subjectivity of interpretation [23]. In multicenter clinical trial settings, inter-observer variability generates cascading methodological and economic challenges. A false-negative interpretation excludes a theoretically eligible patient from the trial, forcing the Contract Research Organization (CRO) to extend the screening phase and activate additional clinical centers, significantly increasing costs. Conversely, a false-positive interpretation enrolls a patient lacking the receptor target necessary to respond to the investigational drug. This scenario is particularly problematic: statistically, non-responders lower the cohort’s overall efficacy average, causing dilution of the effect size. This phenomenon may lead regulatory agencies to reject the drug dossier due to failure to demonstrate statistical superiority over the standard of care. The integration of Deep Learning algorithms for centralized pre-enrollment screening offers a structural solution to this problem. Artificial intelligence systems can automate cellular segmentation across the entire slide, calculating complex metrics—such as the Combined Positive Score (CPS)—in a continuous, objective, and mathematically reproducible manner [3]. This standardization ensures that Sponsors create phenotypically homogeneous cohorts, potentially increasing the statistical power of the study and ensuring that the drug is tested precisely on the biology for which it was designed [22].

### 4.2. Transformation of Central Review and Logistical Optimization

Quality Assurance protocols for multinational clinical trials typically require local eligibility diagnoses, performed at the patient’s originating hospital, to be confirmed by an independent committee of experts (Central Pathology Review). Historically, this process involved physically packaging and shipping slides by courier from peripheral centers to the CRO’s central laboratory. This analog workflow exposes often irreplaceable biological material to the risk of loss or deterioration and generates an average Turnaround Time (TAT) of several weeks. This delay directly affects oncology patients by postponing the initiation of experimental therapy [24]. The adoption of cloud-based Digital Pathology architectures overcomes these logistical limitations. The peripheral clinical center acquires the WSI image immediately after sectioning and staining, uploading it in real time to distributed servers compliant with healthcare data privacy regulations (such as GDPR and HIPAA). Within this ecosystem, Artificial Intelligence initially performs automated quality control (QC), immediately identifying scanning artifacts, air bubbles, or out-of-focus regions and requesting a new scan before the tissue is archived. Subsequently, pre-trained models apply advanced triage logic: they analyze the entire slide and generate heat maps that isolate Regions of Interest (ROIs) with the highest tumor density or diagnostic complexity. The central reviewer, accessing the platform remotely, is visually guided by the algorithm directly to critical areas. This synergistic approach, known as Human-in-the-loop, combines computational efficiency with human clinical oversight, reducing Central Review decision times from weeks to just a few hours and contributing to accelerating the trial’s overall Time-to-Market.

### 4.3. The Emergence of Spatial Biology and Tumor Microenvironment (TME) Analysis

One of the most promising applications of computational pathology in pharmaceutical development is linked to the analysis of the Tumor Microenvironment (TME) through the principles of Spatial Biology. Research in immuno-oncology has clearly demonstrated that the effectiveness of immune checkpoint inhibitors (such as anti-PD-1/PD-L1 therapies) does not depend solely on the absolute number of immune cells present in a sample but is deeply related to their topological arrangement and spatial interactions with neoplastic cells. Advanced Object Detection and semantic segmentation algorithms make it possible to map the exact (X, Y) coordinates of every tumor-infiltrating lymphocyte (TIL). By applying mathematical models to the resulting spatial graphs (such as Voronoi diagrams or Delaunay triangulations), metrics related to the distance between immune cells and the tumor invasive margin can be extracted [20]. This level of analysis enables the pharmaceutical industry to classify tumor architecture into well-defined functional phenotypes: “immune-inflamed” tumors (with active lymphocytes inside the tumor nest), “immune-excluded” tumors (where the surrounding fibrotic stroma acts as a physical barrier against immune cells), and “immune-desert” tumors. Such topological metrics, impossible to quantify through optical observation alone, are emerging as highly robust predictive biomarkers of treatment response. These findings also guide R&D departments in designing combination therapies specifically aimed at remodeling the stroma to convert resistant tumor phenotypes.

### 4.4. Multimodal Integration and Multi-Omics

The current bottleneck in precision oncology is no longer just analyzing a single H&E slide but rather correlating phenotypic features extracted from pathology AI with genomic (mutations) and transcriptomic data [25]. Moreover, AI-based prediction of genetic alterations (e.g., MSI) often suffers from limited specificity when relying exclusively on single modalities. Consequently, addressing the heterogeneity of multimodal data through the preliminary exploration of multimodal foundation models—integrating pathology, genomics, and clinical data—represents a critical next step to significantly enhance the forward-looking value of computational pathology in clinical research [21].

### 4.5. Quantification of Surrogate Endpoints: The Critical Case of pCR

In the design of oncology clinical trials, particularly in the neoadjuvant setting of breast cancer, the use of surrogate endpoints allows the evaluation of drug efficacy to be accelerated without waiting for long-term overall survival data. One of the most commonly used primary endpoints in this context is Pathological Complete Response (pCR), defined as the strict binary absence of viable residual invasive tumor in the surgical bed and regional lymph nodes following pre-surgical systemic treatment. However, when pCR is not achieved, determining the exact extent of remaining disease through the Residual Cancer Burden (RCB) index becomes critical. In most clinical scenarios, evaluating RCB manually is an inherently challenging task. Following months of chemotherapy or targeted therapy, tumor beds are often profoundly altered, characterized by extensive fibrotic scarring, coagulative necrosis, and marked lymphocytic and macrophagic inflammation. Visually distinguishing rare isolated viable tumor cells within this altered tissue is subject to high rates of inter-pathologist disagreement, with frequent risks of overestimating or underestimating the residual disease [26]. The implementation of Deep Learning algorithms specifically trained to identify residual tumor cellularity offers a potential solution to this methodological challenge [27]. AI’s ability to separate necrotic debris from viable tissue and quantify the exact area of residual cellularity provides research organizations and regulatory authorities (such as the FDA and EMA) with an objective, continuous, and permanently verifiable clinical measure. This standardization protects the validity of clinical datasets from subjective human interpretation and ensures a high degree of auditability during rigorous governmental compliance inspections.

## 5. The Strategic Role of Medical Affairs in the Adoption of Digital Pathology

**Figure 1:**
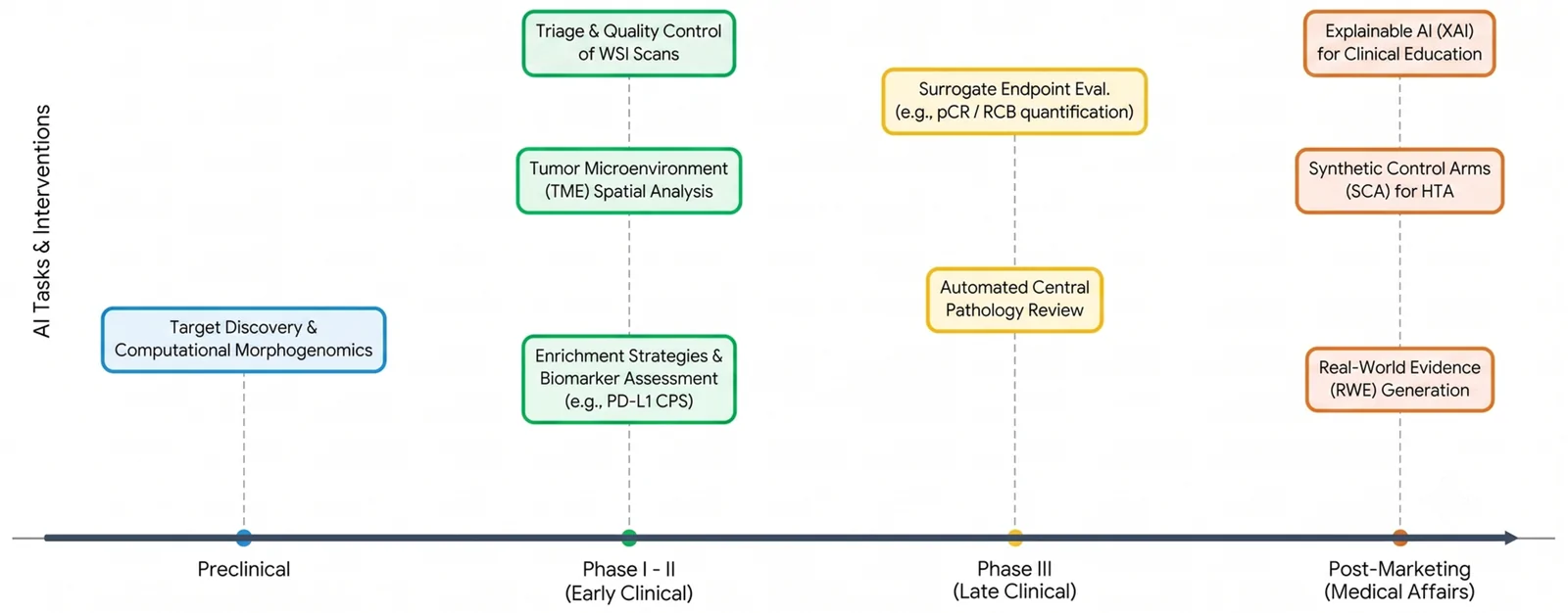
Strategic Integration of Artificial Intelligence across the Biopharmaceutical Value Chain.

While Clinical Research is responsible for certifying a drug’s efficacy and safety within the tightly controlled environment of experimental trials, it is the Medical Affairs function that must ensure that such innovation is properly understood, accepted, and integrated into the complex reality of hospital clinical practice. In the context of precision oncology, this function has evolved beyond its traditional role of scientific communication to assume strategic translational significance, promoting a true operational evolution. As illustrated in Figure 1, Medical Affairs now operates as an essential interface between the development of advanced Data Science solutions and the real needs of prescribing physicians, facilitating the adoption of complex technologies and ensuring their appropriate use [28].

The schematic timeline illustrates the progressive implementation of computational pathology and AI-driven applications at critical stages of the drug life cycle. Along the horizontal axis, the core phases of pharmaceutical development are outlined, from early discovery to post-launch Medical Affairs. The vertical mapping details the corresponding technological interventions: (1) Preclinical Phase: AI accelerates target discovery and computational morphogenomics to infer genotypic alterations from H&E morphology. (2) Early Clinical (Phase I-II): Deep learning models optimize cohort enrichment via standardized biomarker assessment (e.g., PD-L1 CPS), extract topological metrics from the Tumor Microenvironment (TME), and perform automated triage of WSI scans. (3) Late Clinical (Phase III): AI architectures standardize automated central pathology review and provide objective, auditable quantification of crucial surrogate endpoints, such as Pathological Complete Response (pCR) and Residual Cancer Burden (RCB). (4) Post-Marketing (Medical Affairs): Advanced algorithms process large real-world datasets to generate Real-World Evidence (RWE), facilitate the design of Synthetic Control Arms (SCA) for Health Technology Assessment (HTA) submissions, and leverage Explainable AI (XAI) interfaces to support clinical education and physician adoption.

Abbreviations: CPS, Combined Positive Score; HTA, Health Technology Assessment; pCR, Pathological Complete Response; RCB, Residual Cancer Burden; RWE, Real-World Evidence; SCA, Synthetic Control Arms; TME, Tumor Microenvironment; WSI, Whole Slide Imaging; XAI, Explainable AI.

### 5.1. Demystifying the Algorithm: Clinical Education Through Explainable AI (XAI)

The primary barrier to the clinical adoption of diagnostic Artificial Intelligence is not technical or infrastructural in nature, but cognitive. This obstacle is known in the literature as the Black Box problem. Contemporary Deep Learning models process data through multiple neural layers and non-linear mathematical transformations that elude the direct logical understanding of the human operator. Although these systems routinely demonstrate extremely high classification and prognostic stratification accuracy, they lack an intrinsic explanatory capability [29]. For a medical community trained according to the principles of Evidence-Based Medicine (EBM), where every therapeutic decision must be biologically justifiable and traceable, delegating a patient’s diagnostic assessment to an opaque software system understandably generates distrust. In response to this scenario, the educational approach of Medical Science Liaisons (MSLs) and Medical Advisors focuses on demystifying the technology through the principles of Explainable AI (XAI). The most advanced digital pathology software platforms now integrate post hoc interpretation modules designed to make the machine’s decision-making process intelligible. Tools such as Saliency Maps, Attention Heatmaps, and the Grad-CAM algorithm overlay color-coded heatmaps onto the Whole Slide Image (WSI). These maps highlight cellular clusters, nuclear abnormalities, or stromal architectures that the neural network identified as decisive for formulating the diagnosis [30]. The use of these interfaces during scientific discussion forums—particularly Advisory Boards and interactions with Key Opinion Leaders (KOLs)—has proven to be a crucial turning point for clinical change management [31]. By demonstrating how the areas of highest neural network activation (“hot pixels”) frequently coincide with diagnostic regions of interest already recognized in the medical literature, the MSL can help illustrate that the model’s focus is not entirely arbitrary. Rather, it bases its assessments on morphological entities entirely consistent with conventional anatomical pathology taxonomy. While interpretation tools remain subject to technical limitations, this educational pathway is essential for reducing initial skepticism and consolidating the paradigm of Augmented Intelligence [32]. In this model, the algorithm is not intended to replace the specialist but acts as a supportive screening and quantification tool, ensuring efficiency and reproducibility while preserving the physician as the sole decision-maker responsible for the final clinical synthesis.

### 5.2. The Regulatory Transition: From CDx to SaMD Under the IVDR Framework

The development of targeted therapies is clinically and commercially tied to the diagnostic tests required to identify eligible patients, known as Companion Diagnostics (CDx). With the digitalization of pathology, the very nature of CDx has changed: the transition has been from physical kits based on biochemical reagents to cloud-executed software algorithms. This evolution led to the creation of a specific regulatory category, Software as a Medical Device (SaMD) [33]. In Europe, the transition toward SaMD coincided with the implementation of Regulation (EU) 2017/746 on In Vitro Diagnostic Medical Devices (IVDR). Compared with the previous regulatory framework, the IVDR significantly increased the requirements for demonstrating clinical evidence [34]. Currently, most AI-based oncology software is classified within high-risk categories (Class C or D) [35], making the involvement of Notified Bodies mandatory for obtaining CE marking [36]. This new regulatory framework requires Medical Affairs, in close collaboration with Regulatory Affairs departments, to manage significant technical challenges such as continuous model validation. At the center of the debate is the distinction between locked and adaptive algorithms [37]. While the former have parameters fixed at the time of commercialization—ensuring regulatory stability but risking reduced effectiveness when laboratory protocols change—the latter continuously update themselves by analyzing new data (continuous learning). Although adaptive models promise superior long-term performance, their dynamic nature presents complex regulatory challenges. To ensure compliance, medical departments are required to design and fund robust Post-Market Performance Follow-up (PMPF) plans. These plans involve the systematic collection of real-world software usage data from hospitals in order to demonstrate to certification bodies that accuracy, specificity, and clinical sensitivity metrics remain stable or improve over time.

### 5.3. Real-World Evidence (RWE) Generation and Synthetic Control Arms

The progressive digitalization of pathology departments and the structural integration of Whole Slide Images (WSIs) with Electronic Health Records (EHRs) have generated repositories of Real-World Data (RWD) with exceptional scale and granularity. For Medical Affairs departments, the application of computational algorithms to these biobanks represents a crucial tool for generating large-scale Real-World Evidence (RWE). On one hand, this vast amount of data enables extensive retrospective studies aimed at identifying previously unexplored correlations between specific tissue phenotypes and long-term clinical outcomes. On the other hand, it enables a highly innovative methodological advancement: the design of Synthetic Control Arms (SCAs). In the clinical development of therapies intended for ultra-rare diseases, pediatric cancers, or conditions characterized by extremely rapid progression, the use of a traditional control arm—in which patients receive a placebo or suboptimal standard of care—raises profound ethical dilemmas and logistical obstacles that are often insurmountable. In these complex scenarios, biopharmaceutical companies increasingly rely on the synergistic use of Computer Vision systems on histological specimens and Natural Language Processing (NLP) on textual pathology reports to retrospectively identify cohorts of historical patients. Through the combined analysis of this information, it becomes possible to construct control groups whose anatomical-pathological, demographic, and genomic characteristics are clinically comparable to those of the population enrolled in the experimental trial [38]. To ensure the statistical validity of these virtual cohorts, rigorous balancing methodologies are applied, foremost among them Propensity Score Matching [39]. However, it is crucial to note that Propensity Score Matching alone does not entirely eliminate bias. The generation of SCAs remains vulnerable to unmeasured confounding variables, missing EHR data, inconsistencies in endpoint definitions between historical and trial cohorts, and selection bias driven by the variable availability of historical glass slides across different institutions. Despite these limitations, when rigorously designed, this methodological approach generates promising supportive evidence, which is valuable for Health Economics and Outcomes Research (HEOR) departments. The objective demonstration of the therapeutic value and cost-effectiveness of a new molecule compared with highly characterized historical controls is now a fundamental component of submissions to Health Technology Assessment (HTA) agencies, supporting and guiding the complex negotiations required to obtain Pricing and Reimbursement (P&R) approval at both national and European levels [40].

### 5.4. Data Ethics, Data Governance, and Algorithmic Fairness

A further strategic responsibility of Medical Affairs departments is ensuring the ethical sustainability and fairness of algorithmic solutions (Algorithmic Fairness). Scientific literature has shown that diagnostic models trained predominantly on cohorts of Caucasian patients or data originating from high-resource academic centers tend to internalize latent biases related to demographics, genetics, and local laboratory practices [5,41]. Applying a model that lacks sufficient diversity to populations with different phenotypic characteristics or within hospital settings that use less standardized staining protocols carries the risk of a significant reduction in predictive accuracy. Promoting the global adoption of algorithms affected by such distortions risks compromising clinical reliability and exacerbating inequalities in access to precision therapies. Consequently, Medical Affairs has a scientific duty to request and support rigorous external validation studies on multi-ethnic and multicenter cohorts before approving the integration of a partner software solution into regional care pathways. At the same time, promoting Federated Learning architectures—a distributed training method that allows models to be trained directly on local hospital servers without centralizing raw data—represents a key technological solution [42,43]. However, it must be acknowledged that Federated Learning is not inherently a cryptographic technology and does not guarantee absolute privacy, as model updates may still carry reverse-engineering risks without additional safeguards. Furthermore, while it addresses data localization, it does not solve underlying issues of poor data quality, inconsistent slide labels, or unequal site representation.

## 6. Real-World Applications: Case Studies and Strategic Partnerships

The theoretical foundations and engineering architectures of Artificial Intelligence applied to histopathology find their primary validation when integrated into the operational dynamics of the pharmaceutical industry and clinical practice. It is therefore necessary to examine their real-world applications by analyzing already validated and reimbursed algorithms, emerging frontiers in genomic prediction, and the new business models redefining the balance of power in the advanced diagnostics market.

### 6.1. The “Counting Burden” Challenge and Algorithmic Automation in PD-L1 Assessment

The introduction of immunotherapy based on immune checkpoint inhibitors (ICIs) has altered the therapeutic algorithm of numerous cancers, including Non-Small Cell Lung Cancer (NSCLC), urothelial tumors, and gastric carcinomas. The prescription of monoclonal antibodies such as pembrolizumab or nivolumab depends on quantifying the expression of the PD-L1 protein in tumor biopsy tissue. Diagnostically, this parameter is evaluated using complex metrics such as the Tumor Proportion Score (TPS) or the Combined Positive Score (CPS). The CPS, in particular, requires an extremely demanding analytical task: the pathologist must differentiate and count not only tumor epithelial cells positive for the marker but also macrophages and infiltrating lymphocytes dispersed throughout the stroma [44]. This operation creates a substantial visual and cognitive workload (the so-called counting burden) that quickly leads to operator fatigue. Since it is physically impossible for the human eye to accurately count tens of thousands of cells within a few minutes, the assessment ultimately becomes a visual estimate. Near critical clinical decision thresholds (typically 1% or 50%), diagnostic concordance among specialists declines significantly. Classification errors at this stage may either deny patients access to life-saving therapies or expose them to ineffective and potentially toxic treatments. The use of Digital Pathology software platforms (SaMDs) resolves this structural challenge. Algorithms segment the digital slide pixel by pixel, excluding necrotic regions and cutting artifacts, and classify every individual cell (tumor and immune), producing a mathematically derived and reproducible CPS. Independent clinical studies that retrospectively recalculated validation cohort slides using AI have demonstrated that NSCLC patients stratified through algorithmic approaches exhibit statistically superior Objective Response Rates (ORR) and Progression-Free Survival (PFS) compared with those assessed solely through manual interpretation [45]. For pharmaceutical companies, supporting the adoption of these systems in hospital laboratories means optimizing the identification of eligible patients and ensuring that therapeutic indications are applied with maximum fidelity.

### 6.2. From Morphology to Genomics: Direct Mutation Prediction from H&E Slides

While the automation of known biomarker assessment (such as PD-L1 or HER2) represents the refinement of existing workflows, the most innovative frontier in computational pathology is “Computational Morphogenomics.” This research field explores the ability of Deep Learning to infer the genotype of a neoplasm solely through analysis of its morphological phenotype. Currently, identifying actionable mutations (such as EGFR or ALK alterations in lung cancer or Microsatellite Instability—MSI—in gastrointestinal tumors) requires Next-Generation Sequencing (NGS). Although NGS remains the gold standard, it entails significant operational limitations: high costs, the need for sufficient tissue quantities (often scarce in small biopsies), and reporting times that may extend over several weeks, delaying the initiation of targeted therapies in metastatic patients. Recent studies have shown that Convolutional Neural Networks, when trained on large databases of paired omics and imaging data (such as The Cancer Genome Atlas—TCGA), can predict the presence of specific genomic mutations simply by analyzing histological slides stained with Hematoxylin and Eosin (H&E) [19,46]. Since the algorithm cannot directly observe DNA at standard optical resolution, it learns to detect secondary morphological alterations induced by the mutation: subtle changes in chromatin texture, abnormalities in glandular architecture, or characteristic patterns in the topological distribution of inflammatory infiltrates. The translational implications of this technology are substantial. It enables the use of algorithms as rapid, low-cost screening and triage tools: the baseline slide can be analyzed on the same day as the biopsy, and if the AI indicates a high probability of mutation, the sample can be prioritized for confirmatory NGS testing. This strategy has the potential to optimize laboratory resources and significantly accelerate diagnostic and therapeutic pathways.

### 6.3. Critical Assessment of Morphogenomics

While the prediction of genetic alterations directly from H&E slides represents a theoretical paradigm shift, a critical analysis of recent literature reveals significant clinical limitations. Studies often report highly variable Area Under the Curve (AUC) metrics when tested on external validation cohorts, exposing a severe lack of generalizability [47]. The primary weakness of these models is their relatively low specificity; algorithms frequently detect spurious morphological correlations that do not reflect true biological causation [48]. Consequently, rather than viewing these AI tools as a replacement for Next-Generation Sequencing (NGS), the scientific community must critically reposition them as probabilistic triage tools. A negative AI prediction should not preclude molecular testing, but a positive prediction could expedite targeted NGS panels, optimizing laboratory resource allocation.

### 6.4. Strategic Alliances and New Industrial Models (Pharma-Tech Synergy)

The development and large-scale validation of predictive algorithmic models require high-performance computing infrastructures, extensive annotated datasets, and advanced engineering expertise. These requirements often fall outside the traditional competency scope of biopharmaceutical Research and Development (R&D) divisions. This skills gap has encouraged the emergence of strategic alliances, joint ventures, and acquisitions between multinational pharmaceutical companies and technology firms specializing in Data Science and computational pathology [49]. Within this ecosystem, leading pharmaceutical companies have established collaborations with organizations such as Owkin, Paige, and PathAI. The operational model underlying these partnerships is based on complementary assets: pharmaceutical companies provide access to historical digital biobanks enriched with clinical metadata and long-term survival data derived from clinical trials (including unsuccessful ones). Technology companies apply Machine Learning models and Transformer architectures to these datasets to identify novel predictive patterns. If retrospective analysis identifies a morphological signature capable of retrospectively distinguishing the specific subgroup of patients who responded to a drug originally deemed unsuccessful, the molecule may be reintroduced into clinical development for that niche population. In such scenarios, commercial agreements frequently provide for milestone payments and royalties on future sales to the technology company, defining an increasingly hybrid and data-driven R&D model.

### 6.5. The Regulatory Benchmark: Analysis of the “Paige Prostate” Registration Dossier

The integration of AI into clinical histopathology reached a major regulatory milestone in September 2021, when the U.S. Food and Drug Administration (FDA) granted the first De Novo authorization to a neural network-based diagnostic software system for primary pathology: Paige Prostate [50]. Developed using the digital archive of Memorial Sloan Kettering Cancer Center (MSKCC), the software was designed to automatically identify suspicious foci of prostate carcinoma in needle biopsies, operating in the background as a support tool for pathologists. The significance of this approval for Medical Affairs and Regulatory Affairs departments lies in the rigor of the clinical registration study design required by the federal agency to demonstrate safety and effectiveness. Validation was based on a robust multicenter retrospective diagnostic study employing a Multi-Reader, Multi-Case (MRMC) design. Sixteen general pathologists were tasked with evaluating hundreds of challenging slides containing benign mimickers, first without technological support (to establish the human baseline) and later, after a washout period, with the assistance of the software’s visual alerts. The results demonstrated that use of the algorithmic system significantly reduced false negatives, increasing average diagnostic sensitivity by 7.3% without causing a statistically significant loss of specificity. The authorization of the Paige Prostate dossier represents not merely a success story for a single product but the establishment of a methodological benchmark for how the industry must structure validation studies for future oncology Software as a Medical Device (SaMD) products. It defined standards for data management, bias mitigation, and uncertainty quantification—requirements that are indispensable for bringing digital diagnostic technologies to market within an increasingly regulated global healthcare environment.

## 7. Next-Generation AI Architectures: Foundation Models, Vision-Language Models, and LLMs

Despite the success of task-specific Convolutional Neural Networks (CNNs), the current paradigm of computational pathology is often limited by a critical structural flaw: the reliance on supervised learning. Training models for specific diagnostic tasks (e.g., identifying PD-L1 positivity or segmenting glomeruli) requires massive datasets meticulously annotated by expert pathologists. This “annotation bottleneck” makes scaling AI to rare diseases or novel biomarkers prohibitively expensive and prone to subjective human biases (the “ground-truth problem”).

To overcome these limitations, the field is rapidly shifting toward Self-Supervised Learning (SSL) and Pathology Foundation Models. Unlike traditional models, Foundation Models (such as Vision Transformers—ViTs) are pre-trained on millions of unannotated Whole Slide Images (WSIs) [51]. Through SSL, the algorithm learns the fundamental, universal language of tissue morphology by recognizing structural patterns without explicit human labels. Once this generalist representation is acquired, the model can be fine-tuned for a wide variety of downstream clinical tasks using only a fraction of the annotated data previously required [6]. However, a critical limitation remains: these models are computationally intensive, requiring high-performance computing infrastructures that are out of reach for most standard hospital IT departments.

Furthermore, the integration of Large Language Models (LLMs) and Vision-Language Models (VLMs) is redefining the concept of diagnostic synthesis. While visual models analyze the WSI, LLMs can process unstructured text from complex clinical narratives, genomic reports, and historical patient data. VLMs bridge this gap by intrinsically linking histological features with textual descriptions, enabling “zero-shot” classification [52]. For Medical Affairs and HEOR departments, this multimodal synergy is disruptive: algorithms can now automatically generate preliminary pathology reports or dynamically query institutional databases (e.g., “find all historical cases with immune-desert phenotypes and specific EGFR mutations”) to instantly build Synthetic Control Arms. Nevertheless, the clinical adoption of LLMs faces substantial hurdles, primarily the phenomenon of “algorithmic hallucinations”—the generation of clinically plausible but factually incorrect diagnostic texts—which necessitates stringent regulatory safeguards and human-in-the-loop verification protocols.

To fully understand the translational landscape of computational pathology, a critical comparison of the strengths, limitations, clinical readiness, and computational demands of these distinct AI methodologies is summarized in Table 2.

## 8. Conclusions and Future Perspectives: The Strategic Pharmaceutical Horizon

This work demonstrates that while the integration of Artificial Intelligence and Digital Pathology offers transformative potential for clinical research and Medical Affairs, the narrative surrounding its clinical deployment is often overly optimistic. AI has proven its value in optimizing trial recruitment, standardizing biomarker scoring, and generating high-dimensional Real-World Evidence. However, the transition from proof-of-concept algorithms to universally deployable clinical tools is still hindered by fundamental unresolved challenges.

Foremost among these barriers is the vulnerability of current models to domain shift and algorithmic bias. Diagnostic algorithms frequently fail to generalize across different laboratories due to pre-analytical variations in tissue staining and digital scanner calibration. To prevent the exacerbation of healthcare disparities, the recent literature emphasizes that regulatory bodies must mandate multi-institutional and multi-ethnic validation protocols as a prerequisite for Software as a Medical Device (SaMD) approval [30,41]. In this context, prioritizing robust Federated Learning architectures is emerging as the most viable strategy to train global models collaboratively, capturing diverse phenotypic manifestations while strictly preserving institutional data privacy [42,43].

Furthermore, overcoming the historical “annotation bottleneck” requires a definitive methodological transition from Supervised to Self-Supervised Learning (SSL). As highlighted by several breakthrough studies published between 2023 and 2024, the industry is rapidly pivoting toward Pathology Foundation Models [53]. By pre-training on millions of unlabeled Whole Slide Images (WSIs), these advanced architectures reduce the reliance on subjective human annotations. This paradigm shift breaks the “ground-truth glass ceiling,” allowing algorithms to autonomously discover novel prognostic morphological signatures rather than merely replicating the visual limitations and biases of the human eye.

The future of precision oncology, however, lies beyond the isolated analysis of the H&E slide. The most recent technological frontier is represented by early-fusion multimodal architectures. Maximizing AI’s predictive value now requires seamlessly integrating computational histology with spatial transcriptomics, Next-Generation Sequencing (NGS) data, and unstructured Electronic Health Records (EHRs) through the use of Vision-Language Models (VLMs) and Large Language Models (LLMs) [25,52].

Finally, realizing this fully integrated, multimodal ecosystem necessitates a profound evolution in current regulatory frameworks. The rigid, “locked-algorithm” approach mandated by current directives, such as the European IVDR, is intrinsically incompatible with the continuous learning potential of modern AI [34]. Medical Affairs departments must actively collaborate with regulatory agencies to define safe, adaptive lifecycle management frameworks that allow models to safely improve over time based on real-world continuous data streams [37].

In conclusion, the strategic pharmaceutical horizon is not the complete automation of the pathologist but the establishment of a collaborative human-AI diagnostic paradigm [32]. By moving beyond isolated image analysis and embracing multimodal, foundation-level data integration [25,53], the biopharmaceutical industry can effectively transform digital pathology from a descriptive diagnostic support tool into a predictive, critical gatekeeper for precision medicine.

## Figures and Tables

**Table 1:** Key Research in Computational Pathology & AI. This table provides a comparative overview of the foundational studies driving the integration of Artificial Intelligence in digital diagnostics and precision oncology. It outlines the specific clinical applications, the underlying computational methodologies (e.g., Convolutional Neural Networks, Multiple Instance Learning, Multimodal Deep Learning), and their key technical innovations. Crucially, the analysis highlights the structural limitations, domain shift vulnerabilities, and generalization barriers that currently hinder the seamless translation of these advanced algorithms into robust, real-world clinical workflows.

Author and year (Ref)	Clinical Appliction/ Objective	AI Architecture/Methodology	Key Strengths/Innovation	Clinical Limitations & Remaining Challenges
Coudray et al, 2018 [19]	Mutation prediction (EGFR, STK11) directly from H&E slides in NSCLC	Deep Convolutional Neural Networks (Inception-v3)	Proved the concept of computational morphogenomics avoiding costly NGS for preliminary screening	Low specificity and high false positive rates; conflicting findings in validation cohorts; cannot replace molecular testing
Saltz et al, 2018 [20]	Spatial organization of tumor-infiltrating lymphocytes (TILs)	Object detection and spatial correlation networks	Extracted topological feaures (TME) impossible to quantify visually; strong prognostic value	Biological complexity of the stroma is oversimplified; lacks integration with multiplexed immunofluorescence data
Campanella et al, 2019 [6]	Clinical-grade prostate, skin, and breast cancer detection	Multiple Instance Learning (MIL) on 44k WSis	Overcame the ”pixel level annotation bottleneck”; demonstrated high scalability	”Black-box” nature at the patch level; requires massive data sets to generalise; limited fine grained interpretability
Pantanowitz et al, 2020 [2]	Prostate cancer diagnosis on core needle biopsies	Supervised Deep Learnning (CNN)	High sensitivity and real-world validation; foundational for Paige Prostate FDA De Novo authorization	Generalizability drop (domain shift) across different scanners and lab staining protocols remains a structural challenge
Chen et al, 2022 [21]	Pan-cancer integrative histology-genomic analysis	Multimodal Deep Learning (Early and Late Fusion)	Demonstrating superiority of combining WSI and molecular data over single-modality approaches	Extremely high computational cost; missing data modalities in Real-World EHRs severely limit clinical development

Abbreviations: CNN, Convolutional Neural Network; EHR, Electronic Health Record; FDA, Food and Drug Administration; MIL, Multiple Instance Learning; NGS, Next-Generation Sequencing; NSCLC, Non-Small Cell Lung Cancer; TIL, Tumor-Infiltrating Lymphocyte; TME, Tumor Microenvironment; WSI, Whole Slide Image.

**Table 2:** Critical comparison of AI architectures in computational pathology. This table summarizes the fundamental Machine Learning and Deep Learning paradigms currently shaping digital histology. For each architectural model, the core methodological strengths, primary translational weaknesses, current level of clinical readiness (ranging from R&D exploration to commercial regulatory approval), and infrastructural computational demands are outlined.

AI Architecture	Key Strengths	Primary Weaknesses	Clinical Readiness	Computational Demand
CNNs (Supervised learning)	Task specific high accuracy Established FDA pathways	”Annotation bottleneck” High domain shift risk	High (Commercial SaMDs)	Moderate (Standard hospital servers)
VITs (Foundation models)	Annotation free (SSL) High adaptability (few shot)	”Black box” opacity Poor interpretability	Low (R&D/Discovery phase)	Extreme (Massive GPU clusters)
Federated Learning (Decentralized)	Enhanced data privacy Mitigates demographic bias	Network latency Data heterogeneity	Moderate (Multicenter trials)	Moderate (Requires stable bandtwidth)

Abbreviations: CNN, Convolutional Neural Network; FDA, Food and Drug Administration; FL, Federated Learning; LLM, Large Language Model; SaMD, Software as a Medical Device; SSL, Self-Supervised Learning; ViT, Vision Transformer; VLM, Vision-Language Model; WSI, Whole Slide Image.

## Data Availability

The original contributions presented in this study are included in the article. Further inquiries can be directed to the corresponding author.

## Abbreviations

ADCs	Antibody-Drug Conjugates
AI	Artificial Intelligence
AUC	Area Under the Curve
CDx	Companion Diagnostics
CNNs	Convolutional Neural Networks
CPS	Combined Positive Score
CRO	Contract Research Organization
DICOM-WSI	Digital Imaging and Communications in Medicine for Whole Slide Imaging
DL	Deep Learning
EBM	Evidence-Based Medicine
EHRs	Electronic Health Records
EMA	European Medicines Agency
FDA	Food and Drug Administration (U.S.)
FFPE	Formalin-Fixed Paraffin-Embedded
FL	Federated Learning
GANs	Generative Adversarial Networks
GDPR	General Data Protection Regulation
H&E	Hematoxylin and Eosin
HEOR	Health Economics and Outcomes Research
HIPAA	Health Insurance Portability and Accountability Act
HTA	Health Technology Assessment
ICIs	Immune Checkpoint Inhibitors
IVDR	In Vitro Diagnostic Medical Devices Regulation
KOLs	Key Opinion Leaders
LLMs	Large Language Models
MeSH	Medical Subject Headings
MIL	Multiple Instance Learning
ML	Machine Learning
MRMC	Multi-Reader, Multi-Case
MSI	Microsatellite Instability
MSKCC	Memorial Sloan Kettering Cancer Center
MSLs	Medical Science Liaisons
NGS	Next-Generation Sequencing
NLP	Natural Language Processing
NSCLC	Non-Small Cell Lung Cancer
ORR	Objective Response Rates
P&R	Pricing and Reimbursement
pCR	Pathological Complete Response
PFS	Progression-Free Survival
PMPF	Post-Market Performance Follow-up
QC	Quality Control
R&D	Research and Development
RCB	Residual Cancer Burden
ROIs	Regions of Interest
RWD	Real-World Data
RWE	Real-World Evidence
SaMD	Software as a Medical Device
SCAs	Synthetic Control Arms
SSL	Self-Supervised Learning
TAT	Turnaround Time
TCGA	The Cancer Genome Atlas
TIL	Tumor-Infiltrating Lymphocyte
TME	Tumor Microenvironment
TPS	Tumor Proportion Score
ViTs	Vision Transformers
VLMs	Vision-Language Models
WSI	Whole Slide Imaging
XAI	Explainable AI
